# Identification of Highly Conserved Putative Developmental Enhancers Bound by SOX3 in Neural Progenitors Using ChIP-Seq

**DOI:** 10.1371/journal.pone.0113361

**Published:** 2014-11-19

**Authors:** Dale McAninch, Paul Thomas

**Affiliations:** Department of Biochemistry, School of Molecular & Biomedical Science and Robinson Research Institute, The University of Adelaide, Adelaide, Australia; Albert Einsten College of Medicine, United States of America

## Abstract

The transcription factor SOX3 is expressed within most neural progenitor (NP) cells of the vertebrate central nervous system (CNS) and is essential for normal brain development in mice and humans. However, despite the widespread expression of *Sox3*, CNS defects in null mice are relatively mild due to functional redundancy with the other SOXB1 sub-group members Sox1 and Sox2. To further understand the molecular function of SOX3, we investigated the genome-wide binding profile of endogenous SOX3 in NP cells using ChIP-seq. SOX3 binding was identified at over 8,000 sites, most of which were intronic or intergeneic and were significantly associated with neurodevelopmental genes. The majority of binding sites were moderately or highly conserved (phastCons scores >0.1 and 0.5, respectively) and included the previously characterised, SOXB1-binding *Nestin* NP cell enhancer. Comparison of SOX3 and published ChIP-Seq data for the co-activator P300 in embryonic brain identified hundreds of highly conserved putative enhancer elements. In addition, we identified a subset of highly conserved putative enhancers for CNS development genes common to SOXB1 members in NP cells, all of which contained the SOX consensus motif (ACAAWR). Together these data implicate SOX3 in the direct regulation of hundreds of NP genes and provide molecular insight into the overlapping roles of SOXB1 proteins in CNS development.

## Introduction

The SOX (Sry-related HMG box) family of transcription factors (TFs) are expressed in most if not all developing tissues and have critical roles in stem/progenitor cell induction, maintenance and differentiation [1], [2] SOX proteins bind to the minor groove of DNA via an HMG box that has at least 50% identity to the founding member SRY and recognise variations of the core consensus sequence AACAAW (W = A or T) [2]–[4]. In vivo, SOX factor binding typically occurs in association with partner proteins, many of which belong to other major TF families including POU-Oct and zinc finger proteins [5].

Twenty SOX genes have been identified in mammals, which have been divided into groups based on their overall sequence homology. *Sox3*, *Sox2* and *Sox1* belong to the SOXB1 subgroup. These genes are expressed in neural progenitor (NP) cells throughout the vertebrate neuroaxis and are generally downregulated during NP differentiation [6]. In vitro and in vivo data indicate that SOX3 acts predominantly as a transcriptional activator, although there is also evidence supporting repressive activity [1], [7], [8]. Enforced expression of SOX3 in neural progenitors (NP) actively represses their differentiation functioning at least in part to repress Notch signalling [9]. Recent data also suggests that SOX3 may function as a pioneer factor through binding to neuronal-specific genes, priming them for subsequent activation by SOX11 [1], [2]. Despite the widespread expression of *Sox3* in the developing CNS, *Sox3* null mice exhibit relatively mild neurodevelopmental defects, which are restricted to the hypothalamic-pituitary axis, the corpus callosum and the hippocampus [10], [11]. CNS deletion of the other SoxB1 genes is also relatively mild [12], [13]. Together, these data, coupled with overexpression analysis, indicate that SOXB1 proteins functionally interchangeable. This is supported by the recent observation that SOX3 binds to 96% of the known SOX2 binding sites within NP cells [2].

The development of ChIP-seq technology in recent years has provided invaluable insight into TF biology [14]–[16]. These data have highlighted the complexity of transcription factor activity by demonstrating TFs can have tens of thousands of binding sites within a single cell population. While it has been known for many years that TFs can act over long distances, a recent RNAPII ChIP-PET study has added to this complexity by providing further evidence for transcription factor mediated interchromsomal interactions [17]. Many TF binding sites are found at enhancers, promoting gene expression through the recruitment of TFs, cofactors (such as CBP/P300) and RNA Polymerase II (RNAPII) while looping DNA to the target promoter [18]. The ENCODE project has identified ∼400,000 putative enhancer regions in human cell lines based on genomic traits including chromatin methylation and acetylation status, evolutionary conservation and TF binding motifs [19]. Given the human and mouse genomes are in the same order of magnitude, it seems likely that there are a similar number of enhancers. By combining existing data for enhancer regions with TF binding site locations identified using ChIP-seq, we can identify putative enhancers for transcription factors such as SOX3, and begin to understand the functional significance of the vast expanses of non-coding genomic regions.

Identifying SOX3 binding sites and enhancers is crucial for complete understanding of the role of SOXB1 proteins in neural development. Here we present a genome-wide analysis of SOX3 binding in NP cells using ChIP-Seq. Through integration of this data with additional existing datasets we provide evidence that SOX3 and its SOXB1 partners activate hundreds of neurodevelopmental genes through binding to evolutionarily conserved sequences located principally within intergeneic regions. We also identify a putative multi-gene transcriptional hub, implicating SOX3 in interchromosal transcriptional regulation.

## Results

### Identification of SOX3 binding sites in Neural Progenitor cells

**Figure 1:**
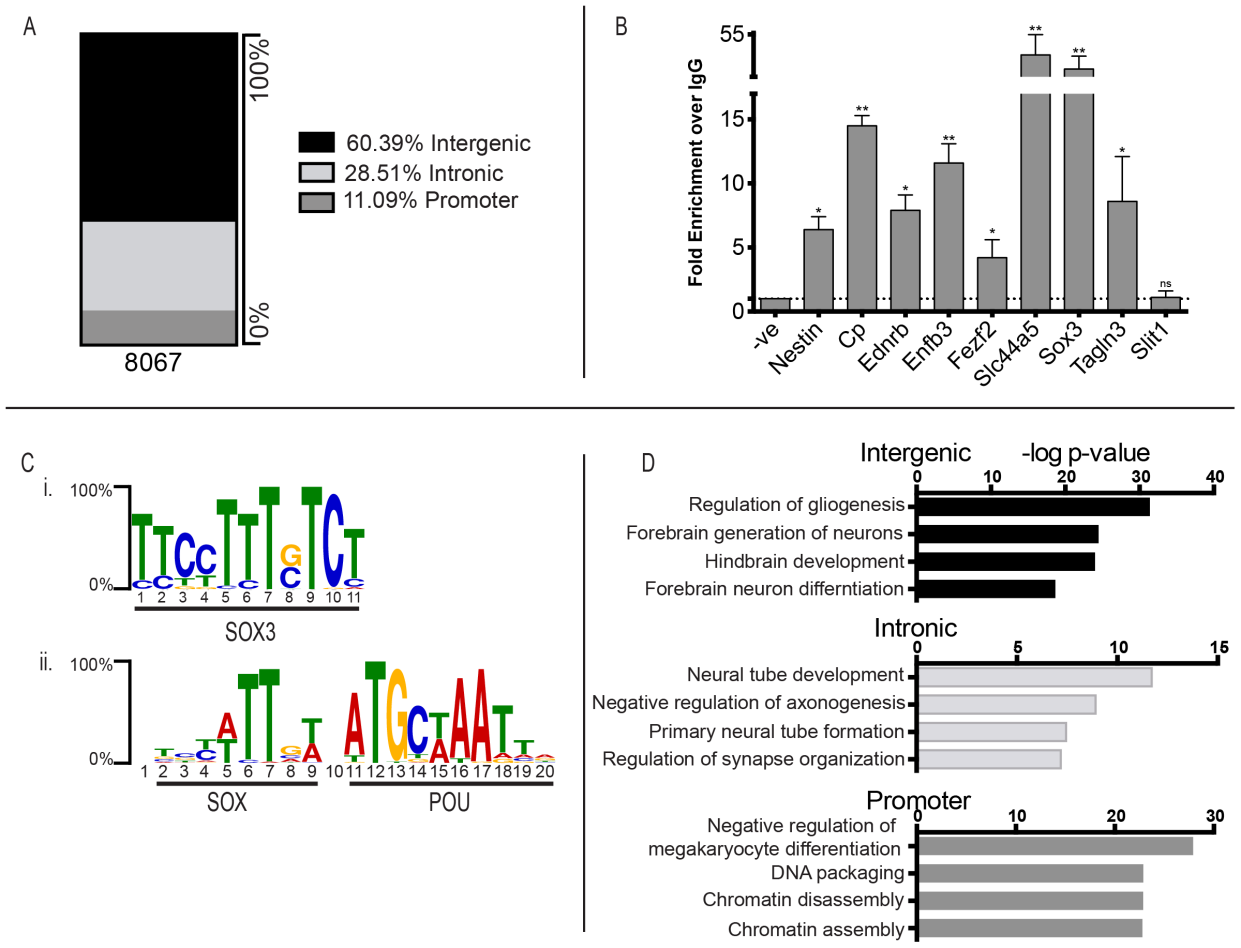
Overview of SOX3 ChIP-Seq data from mouse neural progenitor cells. (A) Genomic classification of SOX3 binding sites relative to nearest transcriptional start sites. (B) Validation of SOX3 ChIP by qPCR. Fold change is relative to both input DNA and IgG control values for the same genomic location. Error bars correspond to standard deviation of three independent sample replicates, P-values indicated as <0.05 (ns), >0.05 (*) and >0.001 (**). (C) Highest enriched DNA motifs identified by MEME-ChIP as i. a SOX motif and ii. a SOX-POU motif. (D) Enriched Gene Ontology terms associated with subsets of SOX3 ChIP peaks.

To identify genomic binding sites of endogenous SOX3 protein, we performed ChIP-Seq analysis of NP cells generated from embryonic stem cells by N2B27 neuroinduction [20]. We have shown previously that these NP cells exhibit robust SOX3 expression [21] and that the SOX3 antibody used for ChIP has specific activity in immunohistochemistry [6] and Western blot analyses [22]. A total of 8067 common binding sites were identified across three independent samples (Figure 1A; Table S1). ChIP-Seq data was validated using ChIP-qPCR on independently generated samples, with all but one of the SOX3 binding sites (SBS) tested showing enrichment (Figure 1B). A *de novo* analysis of the full set of ChIP peaks was performed to identify enriched DNA motifs. Comparison with the JASPAR database [23] confirmed that the most common motif was a SOX binding motif (Figure 1C) (with at least one occurrence within >70% of peaks, p-value less than 10^−6^, and an expected background occurrence of 39%), which was similar to the motif identified in a recently published SOX3 ChIP dataset [2]. The second most common motif features paired SOX/POU binding sites separated by a single nucleotide (Figure 1C) (with at least one occurrence within >40% of peaks). Motifs for other neural TF classes, such as the *Zic, Klf* and *Engrailed* families, were also enriched within 1215, 979 and 647 peaks respectively (all with p-values less than 0.0001). Together, these data indicate successful immunoprecipitation of SOX3-associated chromatin.

Alignment of the peak sequences to the genome revealed that they were associated with 4636 unique nearest neighbouring genes (approximately 20% of genes from the mm9 genome [24]). The majority (60.5%) were located in intergenic regions, 28.5% were located within introns and the remaining 11% were located near the proximal promoter (Figure 1A). GO term analysis revealed that peaks located in intergenic regions were most significantly associated with genes involved in forebrain neuron development (including *Gli3*, *Pax6* and *Wnt8b*) and hindbrain development (including *Hoxa1, Smo* and *Wnt8a*) (Figure 1D; Table S2). Peaks located within intronic regions were highly associated with neural tube development and formation (such as *Shroom3*, *Wnt3* and *Zeb2*). In contrast, peaks located at promoters were significantly associated with genes involved in chromatin modification (including a range of Histone 1, 2 and 4 variants and *Rbbp4*) (Figure 1D; Table S2).

### Conservation of SOX3 binding sites

**Figure 2:**
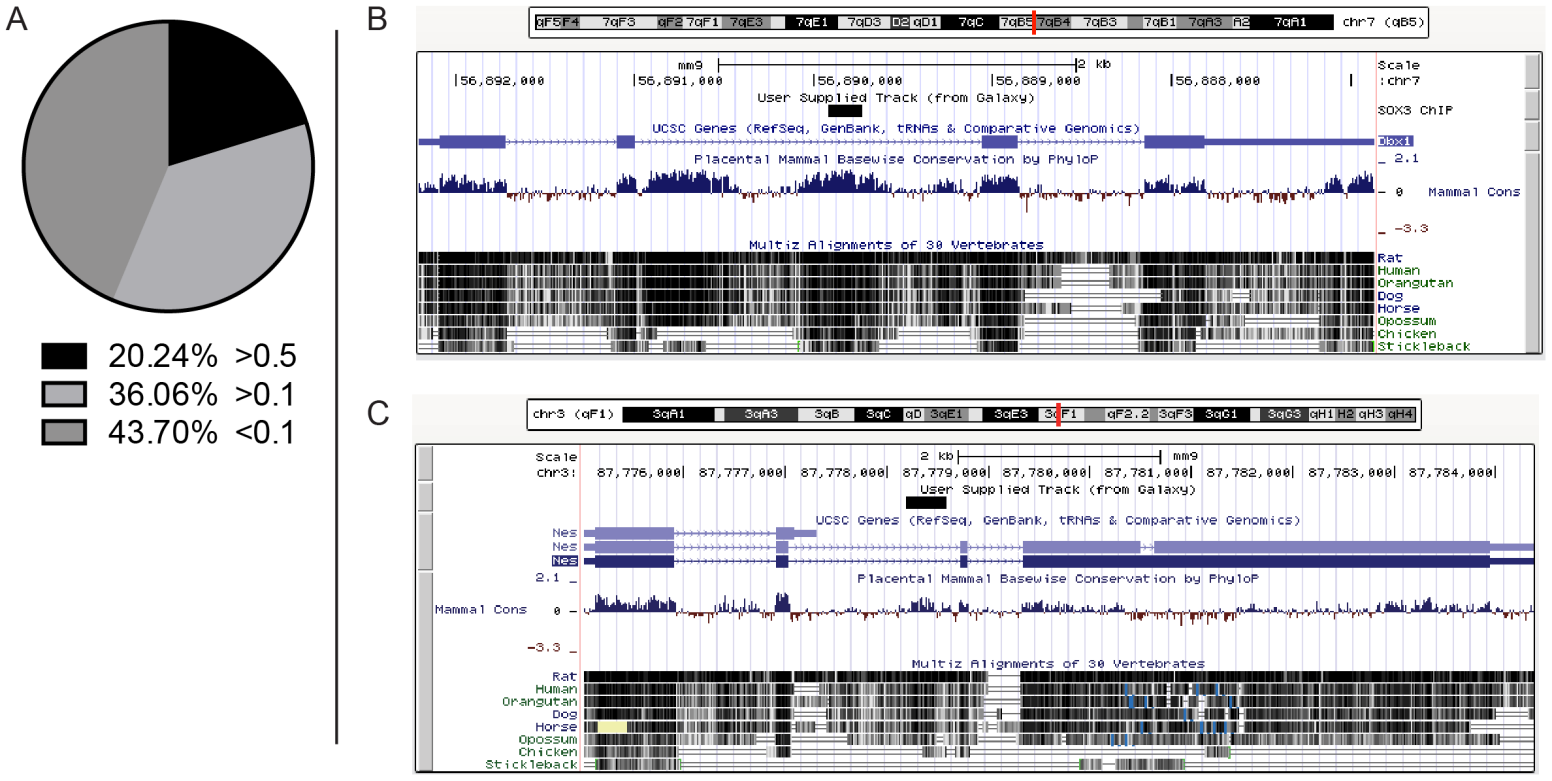
Evolutionary conservation of SOX3 bound regions. (A) The average phastCons score of each SOX3 bound peak showing 20% of peaks are highly conserved across 30 placental mammals. (B) A highly conserved peak within the second intron of *Dbx1* giving the highest conservation score of 0.97 (compared to all peaks). (C) A peak within the second intron of the neural gene *Nestin*, with an average phastCons score of 0.51.

To assess the evolutionary conservation of each peak, we calculated the average phastCons score from data generated from 30 placental mammals provided from the UCSC database. Scores range from 0 to 1 where a value greater than 0.1 shows some conservation between placental mammals, while a value greater than 0.5 is considered highly conserved [25]. The majority (more than 56%) of peaks showed had an average score above 0.1, while more than 20% of peaks exhibited a high level of conservation with a value greater than 0.5 (Figure 2A). When peaks were sorted by genomic location those located in intronic or intergenic regions showed similar levels of conservation (more than 50% above 0.1), while more than 70% of peak located at promoters scored above 0.1. All three locations had a similar percentage of highly conserved peaks scoring more than 0.5 (approximately 20% each). For example, the SOX3 peak within intron 2 of *Dbx1*, a known target of SOX3 [21] has a conservation score of 0.97 and is located within a region of high conservation (Figure 2B). The SOX3 peak within intron 2 of *Nestin*, a well-characterised enhancer shown to bind SOXB1 proteins [26], has an average phastCons score of 0.51 where half the peak is highly conserved while the remaining half is not (Figure 2C).

### Identification of putative neural enhancers that bind SOX3

**Figure 3:**
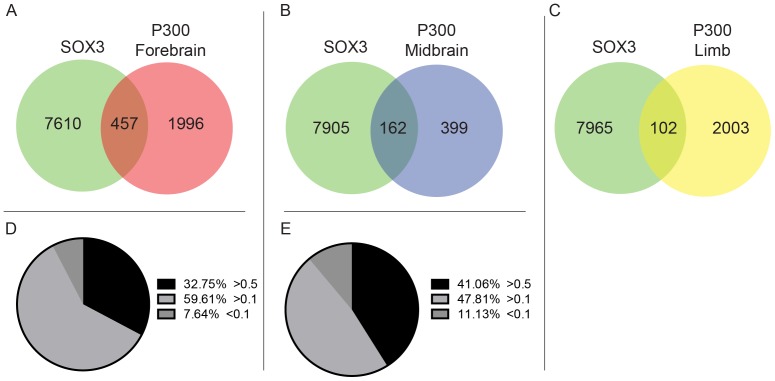
SOX3 binding sites at enhancer regions. The overlap of SOX3 peaks with P300 binding sites identified from 11.5 dpc mouse; forebrain (A), midbrain (B), and limb (C), showing a high degree of overlap in the developing brain and not within the limb. Average phastCons score of the common peaks between SOX3 and P300 forebrain (D) and midbrain (E) binding sites.

To identify possible enhancers bound by SOX3 in NP cells, we overlayed our ChIP-Seq dataset with ChIP-Seq data for the coactivator protein P300 generated from 11.5 dpc embryonic mouse forebrain and midbrain [27]. Peaks were classified as overlapping if the midpoints of each peak were within 300 bp. This comparison revealed that SOX3 bound to approximately 20% and 29% of P300 enhancer regions in the forebrain and midbrain, respectively (Figure 3A, B). Although relatively small, the overlap between these datasets is highly significant, with p-values less than 10^−5^. In contrast, comparison of SOX3 ChIP-seq data with P300 sites from the 11.5 dpc mouse limb bud revealed less that 5% overlap (a non-significant overlap, P<0.6) (Figure 3C). Interestingly, greater than 85% of the common peaks in fore- and midbrain samples had conservation scores above 0.1 while 32% and 41% of fore- and midbrain samples, respectively, were highly conserved (>0.50; Figure 3D, E).

### Identification of conserved SOXB1 binding sites

**Figure 4:**
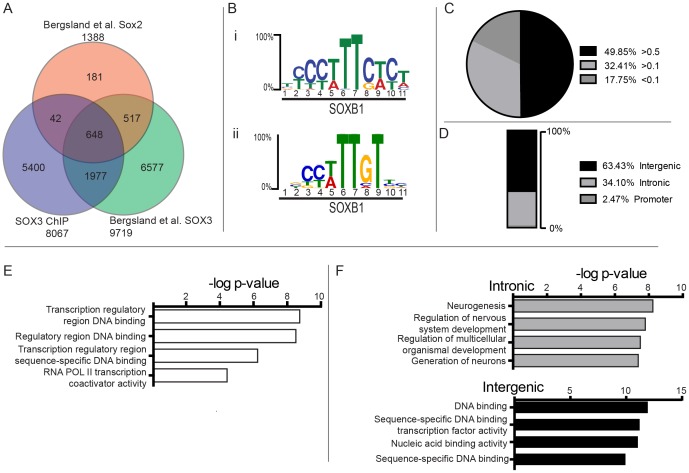
Identification of common SOXB1 regulatory regions. (A) Overlap of this SOX3 ChIP-seq dataset with previously published SOX2 and SOX3 datasets from similar NPCs (2). (B) SOX motif identified with MEME-ChIP present in (i) all 648 SOXB1 common peaks and (ii) 295 peaks. (C) Average phastCons scores for the 648 SOXB1 peaks, showing more than 80% of peaks are either moderately (32%) or highly (42%) conserved. (D) Genomic localisation of the common SOXB1 peaks. Enriched Gene Ontology terms for (E) all 648 SOXB1 peaks and (F) both intronic and intergenic peaks.

Given the overlapping expression and functional redundancy of *Sox1*, *Sox2* and *Sox3*, we next attempted to identify common binding sites for SOXB1 proteins in the developing CNS. Comparison of our SOX3 ChIP-Seq dataset to existing ChIP-Seq data for SOX2 and SOX3 generated from a similar NP cell type [2] revealed 648 binding sites that were common to all three datasets (Figure 4A). Strikingly, MEME-ChIP analysis identified two variants of the SOX consensus motif, one of which is common to all 648 peaks (Figure 4B i), and the second present within 285 peaks (Figure 4B ii). 50% of these peaks showed high conservation, with phastCons scores greater than 0.5, while more than 80% have a score of more than 0.1 (Figure 4C).

Only 2.5% of the SOXB1 binding sites were located in promoter regions, whereas 34% were located within introns and the remaining 63.5% were located within intergenic regions (Figure 4D). GO term enrichment for the 648 SOXB1 peaks indicated that transcription factors were the most common genes regulated by these binding sites (Figure 4E). Intronic sites tend to regulate genes involved in neurogenesis (such as *Fezf2, Robo1*, and *Slit1*) while intergenic sites bind near transcription factors (such as *Irx*, *Nkx*, and *SOX* family members; Figure 4F). Together, these data define a core set of SOXB1 target sites that appear to have evolutionally conserved roles in NP cells.

### Identification of SOX3 binding sites in SOX3 target genes

Although SOXB1 proteins have highly overlapping functions, comparison of genome-wide expression profiles of WT and *Sox3* null NP cells has identified a set of 19 genes with significantly different expression levels, suggesting that a small subset of SOXB1 targets are particularly sensitive to the loss of SOX3 [21]. To investigate whether these genes are direct SOX3 targets, we examined our ChIP-Seq data for binding sites with their intronic and flanking sequences. Thirteen of the 19 differentially expressed genes (68%, with an expected random frequency of 24%, and a p value of less than 0.0001) featured at least one ChIP peak (Table 1), two of which were located at promoters, nine within introns and the remaining seven within intergenic regions. Together, these data suggest that small subset of SOX3 direct target genes require SOX3 (and not other SOXB1 members) for normal expression.

**Table 1:** Differentially expressed genes from *Sox3* null NPCs with nearby SOX3 ChIP binding sites.

Gene	Fold Change	RefSeq ID	Peak Coordinates	Location
*Tmem163*	−1.45	NM_028135	chr1:129472320-129472556	Intron
*Slc44a5*	1.44	NM_001081263	chr3:153836591-153836875	Intron
*Fgfr3*	1.65	NM_008010	chr5:34047895-34048514	Intergenic
*Cpv1*	1.41	NM_025817	chr6:53860009-53860356	Intron
			chr6:53984529-53984835	Intergenic
*Dbx1*	−2.35	NM_001005232	chr7:56889725-56889913	Intron
*Gpr56*	1.56	NM_018882	chr8:97524576-97524923	Intron
*Cspg5*	1.49	NM_013884	chr9:110154883-110154975	Intron
			chr9:110183673-110183904	Intergenic
*Ctgf*	1.45	NM_010217	chr10:24310458-24310740	Promoter
*Flrt2*	1.46	NM_201518	chr12:95662522-95662885	Intergenic
*Ednrb*	1.53	NM_007904	chr14:104243418-104243673	Promoter
			chr14:104298351-104298531	Intergenic
			chr14:104298536-104298789	Intergenic
*Tagln3*	1.54	NM_019754	chr16:45724870-45725203	Promoter
*Slit1*	1.87	NM_015748	chr19:41745438-41745636	Intron
			chr19:41773461-41773782	Intron
			chr19:41790683-41791049	Intron
*Sox3*	−4.10	NM_009237	chrX:57972960-57973254	Intergenic

### SOX3 interaction with an interchromosomal transcriptional network

**Figure 5:**
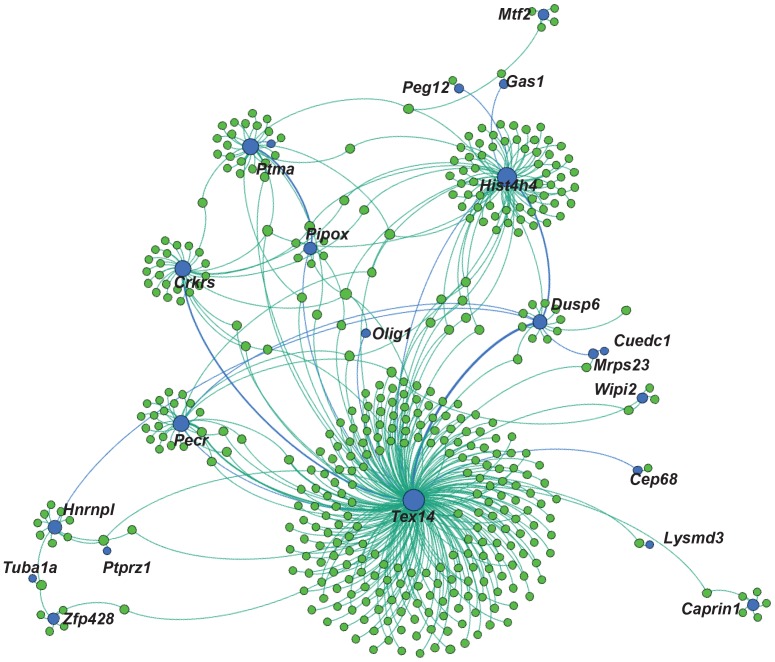
SOX3 is implicated in an interchromosomal regulatory network. Graphical representation of potential long range intra- and interchromosomal interactions as identified by overlap of SOX3 peaks with RNAPII ChIA-PET peaks. Blue circles are sites present in both SOX3 and RNAPII datasets, while green circles are the linked genomic regions present only in the RNAPII dataset. The nearest TSS is labelled for the common binding sites.

A recent study has published a dataset for chromatin interaction analysis with paired end tagging (ChIA-Pet) of RNA polymerase II in neural stem cells [17]. They identified more than 5,000 putative enhancers linked to the promoter of genes on different chromosomes (interchromosomal interactions), as well as more than 10,000 enhancers linked to distant genes on the same chromosome. We sought to identify whether SOX3 could be linked to any of these putative inter- or intra- chromosomal enhancers identified from a neural stem cell population. From the 8067 SOX3 peaks identified, 97 overlapped with potential long-range enhancers (a significant overlap, P<0.001, with an expected overlap of 34 by chance) that can be linked to 304 and 246 inter- and intrachromosomal promoters, respectively. For example, SOX3 binds an intronic enhancer within *Tex14* (Figure 5) that can be linked to 263 different promoters and enhancers. This putative enhancer has a phastCons score of 0.12, moderate evolutionary conservation, and features a single SOX binding site. These data suggest that SOX3 may be involved in complex, long-range gene regulation.

## Discussion

This study has identified 8067 regions within the genome of murine NP cells that are associated with SOX3. The majority of the SOX3 binding sites are not located at the proximal promoter of genes but rather are within intronic or intergenic regions, suggesting an extensive regulatory (enhancer-binding) role for SOX3 in NP cells. Interestingly, the most common motif within these peaks was a variation of the standard SOX DNA binding motif that contained a strong preference for a C residue at position 10 (C_10_) instead of a T (T_10_). Significant enrichment for C_10_ was also identified by Bergsland et al. 2011 using an independent SOX3 antibody, although not to the same degree as in our study. Previous structural studies of SOX2 binding to a T_10_-containing binding site indicate that T_10_ makes contact with arginine 5 of the HMG domain as well as the c-terminal tail. However, as residues within these regions are completely conserved throughout SOXB1 members, it remains unclear why SOX3 prefers to bind the C_10_ sequence *in vivo*. Given that partner factors can influence SOXB1 binding site preference [28], it is possible that the strong enrichment for the C_10_ site may reflect SOX3 partner protein usage in NPCs.

### SOX3-POU cooperation

SOX transcription factors cooperate extensively with specific partner factors, including OCT, BRN, or other SOX family members [29]. Co-factor DNA binding is a common developmental mechanism that provides exquisite temporal and spatial expression of target genes [30]. The second most common motif identified from the complete SOX3 ChIP-Seq dataset (within 1998/8067 peaks) was a combined SOX/POU DNA binding motif (Figure 1). Similar enrichment of this motif was also identified in the SOXB1 subset (126/648 peaks; p value <0.0001) [31]. The nucleotides comprising the POU component of the SOX/POU motif show minimal variation, while the nucleotides comprising the SOX motif show greater variability, suggesting the POU-DNA interaction is more sensitive to sequence composition. To assign functional significance to these binding sites it would be useful to delete these binding sites either completely or each of the SOX and POU binding sites separately [32]. This could provide information as to the importance of these linked binding sites indicating whether both factors required or if one is sufficient.

### Many SOX3 binding sites exist within biologically important enhancers

Although the preferential binding of SOX3 in or near many known neurodevelopmental genes is suggestive of a wide-ranging regulatory role in NP cells, it is difficult to identify the functional significance of these sites from binding data alone. To address this, we assessed the evolutionary conservation of each ChIP peak, with the rationale that a highly conserved peak has the potential to be more biologically relevant if selection pressure has maintained sequence conservation throughout evolution. The proposed link between conservation and function is supported by previous studies that have demonstrated that developmental enhancers can be reliably identified solely on sequence conservation [33], [34]. Our data show over 50% of SOX3 peaks are either moderately (36%) or highly (20%) conserved, having PhastCons scores above 0.1 or 0.5, respectively. Amongst the highly conserved peaks we identified the well-characterised intronic *Nestin* enhancer that has been shown previously to bind SOXB1 proteins *in vitro* and drives NP cell expression of reporter genes in the developing CNS [26]. We also identified a SOX3 peak within the second intron of *Dbx1* that had the highest overall conservation score and has been linked to the expression of *Dbx1* in cultured NPCs and the spinal cord of 9.5 dpc *Sox3* null mice [21]. In addition, SOX3 peaks were identified at independently identified *cis* regulatory motifs (CRM) shown to respond to SOXB1 transcription factors including *Olig2, Dbx2* and *Nkx2.2*, although not at the remaining CRMs identified in this study [35]. Further comparison of our data to ChIP-Seq data for the co-activator P300 from mouse 11.5 dpc forebrain and midbrain, identified 457 (19.85%) and 162 (28.88%) common peaks, respectively. Many of these P300 associated regions have been shown to function as CNS enhancers *in vivo* [27]. Interestingly, overlapping SOX3/P300 peaks showed an increased enrichment for high conservation with >32% (forebrain) and >41% (midbrain) of peaks being highly conserved. Overlapping P300 peaks from the limb also showed high conservation, approximately 42%, which is to be expected given P300's high level of association with enhancers. Although only a small percentage of the total number of SOX3 ChIP peaks, this is only data pertaining to one co-activator (P300), it is likely that other peaks feature alternate co-activators or other proteins required for SOX3 to act as a pioneering factor [2]. Taken together, these data suggest that many SOX3 peaks are likely to correspond to important NP enhancers.

Previous ChIP-Seq analysis of SOX3 in a similar population of NP cells also identified extensive binding across the genome (9719 SOX3 binding sites). Comparison to our dataset identifies more than 15,000 unique SOX3 binding sites, 2625 of which are common to both studies. Although both analyses produced a similar number of binding sites individually, the overlap in binding sites between datasets was lower than anticipated. This variability is likely to be caused by use of different NPCs. Although both studies used NPCs that were generated from ES cells by N2B27 induction [20], the Bergsland et al. culture conditions also included bFGF, SHH, and retinoic acid [2]. These additional factors have major roles in CNS patterning and are therefore likely to influence the identity of the overall NP cell population. It has been observed previously the degree of overlap of binding sites between different cell lines can vary, from less than 50% (SRF peaks from 3 different human cell lines [36]) to more than 80% overlap (E2F4 peaks from multiple primary mouse tissues and cell lines [37]). It is also important to note that different SOX3 antibodies were used in the ChIP-seq studies. Although both have been shown to be specific they likely recognise different SOX3 epitopes, potentially introducing variation in the SOX3 binding sites identified.

Given the functional redundancy of SOXB1 proteins, we sought to identify genomic regions bound by SOX3 and SOX2 in NP cells. The 648 common SOXB1 binding sites showed greater enrichment for conserved peaks than any of the datasets alone with approximately 50% having high conservation. Remarkably, two variants of the SOX motif were identified through *de novo* screening of the 648 SOXB1 peaks. The most common motif was found in all 648 peaks with a highly significant E value (4.7e-371). This motif is very similar to the one identified from the complete SOX3 dataset (although there was no nucleotide preference observed at position 10), and essentially identical across positions 6, 7 and 9 which are known to make contact with the HMG domain [4]. The second SOX motif identified was present in 285 peaks (with an E value of 1.5e-23) and was more similar to the SOX3 motif, with an increased nucleotide preference at position 10. GO term analysis indicated that SOXB1 common peaks bind nearest to transcription factors, in particular members of the SOX family (including *Sox1*, *Sox2, Sox4, Sox5, Sox6, Sox9, Sox11*), *Nkx2.1 and Nkx2.2* all with known roles in neural development. The intronic peaks are enriched for genes involved in neural development, including *Fzd3* and *Fzd6* both of which are required for correct ventricle formation in the developing midbrain [38]. Together these data give evidence that SOXB1 binding sites have been highly conserved throughout evolution and potentially regulate genes important for neural development, and also suggests extensive cross regulation of SOX factors consistent with analyses of individual factors [39], [40].

The expression level of most NP genes is not affected by the loss of *Sox3* [21], presumably due to functional redundancy with other SOXB1 members. However, we have previously identified nineteen genes that have altered expression in *Sox3* null NP cells, suggesting that some genes are particularly sensitive to SOX3 loss of function. Here we show that the majority of these genes (68%) are directly bound by SOX3. The high frequency of binding sites in differentially expressed genes appears significant given that SOX3 binding sites on average are found in only 20% of genes genome wide. The mechanism that underpins the sensitivity of these direct targets to the loss of SOX3 alone is currently unclear. Given that the HMG box sequences of the SOXB1 proteins are not identical, one possibility is that the binding sites in these differentially expressed genes have a higher affinity for SOX3 than other SOXB1 proteins. Alternatively, these genes may be sensitive to the overall dosage of SOXB1 protein rather than SOXB1 itself. Additional studies such as SoxB1 gene swap experiments are required to further investigate this issue.

Finally we have identified a number of potential genome wide interactions, linking enhancers and promoters of genes regulated by SOX3. It was observed that an intronic enhancer within *Tex14* could be linked to many other enhancers and promoters, forming a transcriptional hub. These networks may aid in ensuring transcription of specific genes occurs at similar time, potentially allowing for quicker responses. As noted by Zhang et al. 2013 the RNAPII data identifies regions of pre-initiation events; as such not all genes will be actively transcribed. Further to this, it is likely that there are other long-range interactions that do not involve RNAPII. It could be informative to perform a ChIA-PET against SOX3 in NPCs to see what long-range interactions occur independent of RNAPII. It would also be interesting to see whether deleting one of the binding sites that form part of a transcriptional hub, such as *Tex14*, has an effect on the transcription at connected genomic locations.

## Conclusions

In conclusion, we have shown that SOX3 binds extensively to evolutionarily conserved sequence in or near known neurodevelopmental genes that are likely to function as enhancers. Further functional validation within an *in vivo* system is required to assess the functional importance of these data. With the recent emergence of genome editing tools, CRISPR and TALENs, the generation of genetically modified mice has become significantly more streamlined and efficient. This technology will allow for the simple deletion or modification of binding sites in mice to highlight the importance of each binding site providing biological significance.

## Materials and Methods

### NPC generation

Mouse R1 ES cells, as described previously [10], [22], were passaged without feeders in DMEM (Gibco) in the presence of LIF and foetal calf serum. ES cell monolayers were cultured in N2B27 media for 4 days to produce NPCs as described previously [20].

### SOX3 ChIP-seq

NPCs were fixed in 1% formaldehyde for 8 minutes at room temperature, lysed and sonicated (Bioruptor, Diagenode) for 15 minutes in 1-minute pulses on ice. SOX3 bound chromatin was immunoprecipitated by a goat polyclonal antibody raised against human SOX3 (R&D systems, AF2569). DNA was recovered by reversing crosslinks, and purified by PCR clean-up kit (QIAGEN). Three independent DNA libraries were produced with the Illumina TrueSeq library kit as per manufacturer's instructions, and libraries were sequenced on the Illumina HiSeq producing 50 bp single end reads. A control sample (without SOX3 antibody) was run as input for background control. ChIP samples were validated by qPCR (StepOne Plus, Applied Biosystems) using Fast SYBR (Life Technologies). Signals were considered positive when Ct_sample_ (non-enriched region) - Ct_sample_ (peak region) was >2 following normalisation to Ct_IgG_.

### Peak Calling

Bowtie [41] was used to align reads to the mouse genome (mm9). Peaks were called for each biological replicate using MACS [42], with bandwidth of 300, a model fold of 10–30, using input sample as background control, and a p-value threshold of 1e-5. Only peaks present in all 3 biological replicates were retained.

### Gene ontology

Gene ontology was performed using GREAT (http://great.stanford.edu/) for regions associated with SOX3 bound enhancers [43]. Default ‘basal plus’ parameters were used to define gene locus, and the whole genome was used for background regions.

### *De Novo* motif enrichment

MEME (http://meme.sdsc.edu/) was used to identify DNA enriched motifs from SOX3 bound peaks [44]. WebLogo [45] was used to visualise *de novo* motifs generated by MEME-ChIP.

### Published ChIP-Seq data

ChIP-Seq data on SOX2 and SOX3 in NPCs were obtained from GSE33024, P300 data from GSE10516, ChIA-PET data from GSE44067.

### Primers for qPCR

Primers for validating ChIP samples by qPCR are listed in a 5′ to 3′ direction as follows: *Nes* F- GCCCCAGTCAGTCTTCTGAG, R- GCTGGTGACAGACAAAAGCA,

*Cp* F- CTCACACTGTGCTGGGCTAA, R- AGGAAGTfTGTGCAACTCTGGA,

*Ednrd* F- CTAAACAGGCCTCTCGCAAC, R- TTGTCTGGGGACAGCAAAG,

*Enfb3* F- ATGCTCAGCACCTCATTGG, R- GGCACGTGACTGGTGGTAG,

*Fezf2* F- GGTCGTCTTTTCTTCCTGTCC, R- ATCCACAGAACCAGCATCACT,

*Slc44a5* F- CTGCCTGGATGTCAGGATTT, R- CCCACAGTGTTTGTAGGAACG,

*Sox3* F- ATGAGTTTCCGGAATGTTGC, R- CTTCTCACTTCCTGCCCTTG,

*Tagln3* F- CCTCTCCTAGACAGGCCAGA, R- GTGGGGCCTCAGATACAATG,

*Slit1* F- AGACGGACCTGGGAAATTCT, R- CCAGAAAGCAGGATTTGCAT

## Supporting Information

Table S1 **A list of all SOX3 peaks identified by ChIP-seq.** Featuring mm9 coordinates, nearest gene, RefSeq gene ID and distance from centre of peak to nearest transcriptional start site.
(XLSX)

Table S2 **A list of the top 20 GO terms associated with the intergenic, intronic and promoter SOX3 ChIP-seq peaks.**
(XLS)
